# Considerations for the Full Global Withdrawal of Oral Polio Vaccine After Eradication of Polio

**DOI:** 10.1093/infdis/jix105

**Published:** 2017-07-01

**Authors:** Lee M. Hampton, Gaël Maufras du Châtellier, Jacqueline Fournier-Caruana, Ann Ottosen, Jennifer Rubin, Lisa Menning, Margaret Farrell, Stephanie Shendale, Manish Patel

**Affiliations:** 1 Global Immunization Division, Centers for Disease Control and Prevention, Atlanta, Georgia; 2 West and Central Africa Regional Office, UNICEF, Dakar, Senegal; 3 World Health Organization, Geneva, Switzerland; 4 Supply Division, UNICEF, Copenhagen, Denmark; 5 Program Division, United Nations Children’s Fund (UNICEF), New York, New York; 6 Task Force for Global Health, Atlanta, Georgia

**Keywords:** Oral polio vaccine, polio eradication, vaccine derived polioviruses

## Abstract

Eliminating the risk of polio from vaccine-derived polioviruses is essential for creating a polio-free world, and eliminating that risk will require stopping use of all oral polio vaccines (OPVs) once all types of wild polioviruses have been eradicated. In many ways, the experience with the global switch from trivalent OPV (tOPV) to bivalent OPV (bOPV) can inform the eventual full global withdrawal of OPV. Significant preparation will be needed for a thorough, synchronized, and full withdrawal of OPV, and such preparation would be aided by setting a reasonably firm date for OPV withdrawal as far in advance as possible, ideally at least 24 months. A shorter lead time would provide valuable flexibility for decisions about when to stop use of OPV in the context of uncertainty about whether or not all types of wild polioviruses had been eradicated, but it might increase the cost of OPV withdrawal.

Widespread use of oral polio vaccines (OPVs) since the 1960s has resulted in the eradication of wild poliovirus type 2 (WPV2), the lack of WPV3 detection since November 2012, and the confinement of WPV1 to areas of Afghanistan, Nigeria, and Pakistan by 2016 [1–3]. OPVs are relatively inexpensive and easy to administer and can provide good protection against poliomyelitis and poliovirus infections [3–5]. However, the attenuated polioviruses in OPVs can undergo genetic changes during replication, which, in communities with low vaccination coverage, can result in vaccine-derived polioviruses (VDPVs) that can cause paralytic polio [6]. From January 2006 to May 2016, 721 polio cases were caused by circulating VDPVs (cVDPVs) [6]. Eliminating the risk for polio caused by VDPVs will require stopping use of all OPVs in all routine immunization services and supplementary immunization activities (SIAs).

The first phase in the eventual cessation of all OPV use was the globally synchronized switch (hereafter, “the switch”) from trivalent OPV (tOPV), which contained types 1, 2, and 3 live, attenuated polioviruses, to bivalent OPV (bOPV), which contains only types 1 and 3 attenuated polioviruses. As part of the switch, all countries and territories using OPV officially ceased use of tOPV by May 2016 and withdrew all live, attenuated type 2 polioviruses from vaccine stores at all administrative levels [3, 7–9]. The live, attenuated type 2 polioviruses were prioritized for removal because their use had accounted for >94% of the polio cases from cVDPVs from January 2006 to May 2016, yet no cases of polio caused by WPV2 had been detected since 1999 [6]. Once WPV1 and WPV3 are certified as eradicated, use of bOPV will no longer be required, and it will need to be withdrawn (Table 1). Globally, synchronizing bOPV withdrawal will help prevent the spread of any attenuated polioviruses that could eventually become cVDPVs from countries that continue to use bOPV to countries that have ceased bOPV use and will therefore have increasing population susceptibility to poliovirus infections [10].

**Table 1.** Comparison of the Switch From Trivalent Oral Polio Vaccine (tOPV) to Bivalent OPV (bOPV) Versus the Full Withdrawal of OPVs

Comparison	**Switch From tOPV to bOPV**	**Full Withdrawal of OPVs**
Reason for change	End of transmission of type 2 wild polioviruses made the risks from continued regular use of OPV containing type 2 Sabin strain polioviruses outweigh the benefits	End of transmission of all 3 types of wild polioviruses will make the risks from continued regular use of any OPV containing Sabin strain polioviruses outweigh the benefits
Synchronization	All countries using tOPV needed to withdraw all tOPV in a synchronized manner within a short time frame to avoid creating type 2 cVDPVs	All countries using OPV will need to withdraw all OPV in a synchronized manner within a short time frame to avoid creating cVDPVs
Potential risks from incomplete withdrawal of vaccine	tOPV left in the cold chain and used long after the switch could potentially result in new cVDPVs	OPV left in the cold chain and used long after full OPV withdrawal could potentially result in new cVDPVs
OPV use in routine immunization after event	Routine immunization programs used bOPV instead of tOPV after the switch	No OPV should be used in routine immunization programs after full OPV withdrawal
Introduction of new form of OPV during event	bOPV was introduced simultaneously with the withdrawal of tOPV during the switch	No new form of OPV will be introduced during full OPV withdrawal
Availability of OPV stockpile	Monovalent OPV stockpile available for use in response to polio outbreaks caused by type 2 polioviruses after the switch	Monovalent OPV stockpiles will be available for use in response to polio outbreaks caused by any type of poliovirus after OPV withdrawal
Outbreak response resources	Extensive resources available for organizing responses to polio outbreaks caused by cVDPVs after the switch	Fewer resources may be available for organizing responses to polio outbreaks caused by cVDPVs after full OPV withdrawal

Abbreviation: cVDPV, circulating vaccine-derived poliovirus.

To build on the current stockpile of monovalent type 2 (mOPV2) vaccine established after the switch, stockpiles of monovalent type 1 OPV and type 3 OPV will need to be maintained for responding to any outbreaks of polio that occur after bOPV withdrawal [3, 7]. Any remaining vials or containers of OPV (mOPV, bOPV, or tOPV) that are identified outside of those stockpiles will need to be withdrawn [11–13].

Full OPV withdrawal will also involve stopping the manufacture and distribution of bOPV, destroying all OPV withdrawn from vaccine stores, conducting monitoring to ensure that all OPV outside of the mOPV stockpiles has been successfully withdrawn, and ensuring that sufficient financial and human resources are available for this work (Figure 1). To facilitate timely initial planning for full withdrawal of all OPV vials outside of the mOPV stockpiles, henceforth referred to as “OPV withdrawal,” the Global Polio Eradication Initiative’s (GPEI’s) Immunization Systems Management Group (IMG) discussed how the withdrawal of all OPVs might benefit from the experience gained with the switch at a meeting in September 2016. This article reflects that discussion.

## MANUFACTURING AND DISTRIBUTION OF BIVALENT OPV

**Figure 2.**
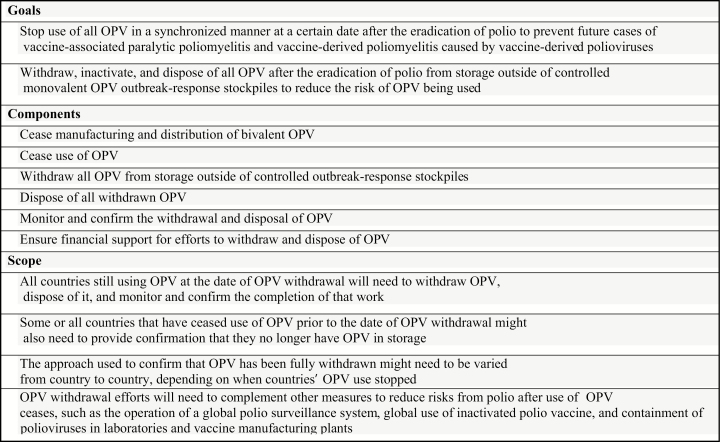
Countries using oral poliovirus vaccine (OPV) in May 2016 following the global switch from trivalent OPV to bivalent OPV. Data are unpublished and from the World Health Organization Immunization Repository.

As of August 2016, 6 companies were producing World Health Organization (WHO)–prequalified bOPV for the international market [14], with 7 additional companies producing bOPV for their domestic markets [15]. Among the 150 countries and territories currently using OPV (Figure 2), approximately 75 procure bOPV through the United Nations Children’s Fund (UNICEF), while the rest self-produce or self-procure bOPV. Among countries and territories procuring bOPV through UNICEF, SIAs are the key demand driver, with routine immunization accounting for approximately 20% of UNICEF’s procurement of 1.2 billion doses in 2016. Similar to tOPV withdrawal, successful withdrawal of bOPV will depend on finding a balance between the scaling back of production by global and domestic manufacturers and ensuring sufficient availability of types 1 and 3 bulk and finished bOPV to meet routine immunization, outbreak response, and planned SIA demand through the date of OPV withdrawal [13]. Factors that can facilitate a favorable balance include advanced notification of the date of withdrawal to manufacturers and countries, inventories of countries’ OPV stocks, and accurate country and global quantification of demand for bOPV. Adequate planning and coordination among UNICEF, other GPEI partner organizations, governments, and manufacturers will be required to ensure that appropriate supplies of both bulk stocks and finished bOPV are available in sufficient quantities to meet global demand [16]. All OPV manufacturers will need to cease production of bOPV well in advance of OPV withdrawal. Except for manufacturers contracted to produce mOPV bulk and finished product for the mOPV emergency use stockpiles, all OPV manufacturers will need to exit the market [3, 17], albeit in a closely monitored and managed fashion that allows sufficient availability of bOPV.

**Figure 1.**
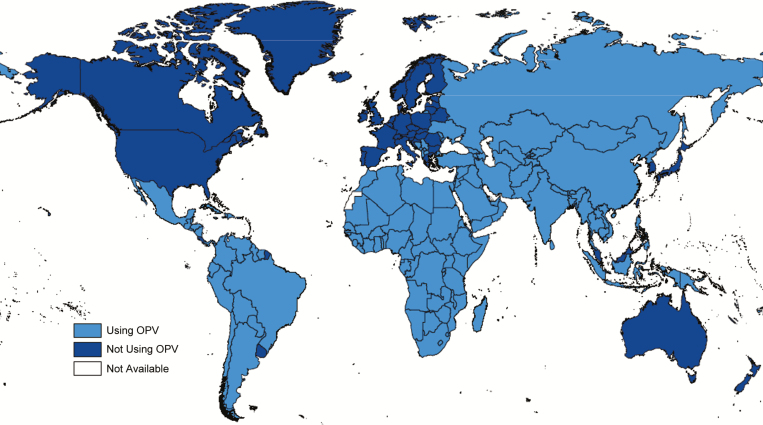
Considerations for Final Withdrawal of Oral Poliovirus Vaccine (OPV), Immunization Systems Management Group, 2016.

As with the switch, a clear commitment from all OPV-using countries that they will cease all bOPV use in a synchronized manner will be required through a World Health Assembly resolution [18]. A clear indication from GPEI as to the likely date of OPV withdrawal, similar to the statement in the 2013–2018 Polio Eradication and Endgame Strategic Plan that the switch would likely occur in mid-2016, would also be useful [7]. Since it can take up to 24 months to produce OPV, manufacturers will require advanced notice of at least 24 months to plan the cessation of bOPV production and avoid having large excess stocks that will need to be destroyed after OPV withdrawal [16].

Procurement policies and stock management systems that minimize the likelihood of stockpiling excessive amounts of bOPV in individual countries can guide procurement decisions and help reduce the amount of bOPV that will need to be destroyed after withdrawal. In particular, each country using bOPV will need to perform a careful inventory of bOPV stocks and plan bOPV requirements and deliveries to ensure that manufacturers have sufficient information on bOPV needs to secure sustainable supply while avoiding overstocks. Given the time it can take to produce OPV, such inventories will be most helpful if they are initially conducted at least 24 months prior to the date of OPV withdrawal. Based on the experience with the switch, early outreach to self-procuring countries regarding such inventories and associated procurement planning would be especially important because many such countries use 2- to 3-year contracts with suppliers. Coordination with self-procuring countries to ensure that there is some flexibility built into their supply contracts could be helpful. Reaching out to private sector immunization providers and vaccine distributors about OPV withdrawal so they can factor it into their procurement planning would also be warranted.

## USE OF OPV

Global guidance for the switch recommended that messaging related to the switch emphasize both the need to stop tOPV use and the need to use bOPV after tOPV use stopped [19]. The global guidance also recommended that such messaging be aimed primarily at immunization program staff, health workers, health-related nongovernment organizations, and other key stakeholders. Given the complexity of the rationale for the switch, communications activities aimed at the general public, including high profile switch-related ceremonies, were not recommended in most contexts. With OPV withdrawal, however, communications messages can be much simpler, focusing on the need to stop all OPV use because all wild polioviruses have been certified as eradicated. This more simple and positive message will be appropriate for public communications and would complement outreach efforts and trainings aimed specifically at healthcare workers and immunization program staff. Several countries have found that very limited tOPV use continued for months after the official switch date, when all tOPV use should have stopped. For example, in Hyderabad and Ahmedabad in India, postswitch tOPV use was found in a small number of private clinics, most of which were very small facilities not affiliated with professional medical organizations [20]. Broader, more direct communications messages both leading up to and following the full withdrawal of OPVs might reduce the likelihood of OPVs being used beyond the global withdrawal of OPVs outside of any needed outbreak responses by helping to reach all clinics and facilities with the necessary information.

Training of immunization and logistics staff at the global, regional, national, and local levels was essential to the success of the switch [8] and will also be needed for OPV withdrawal. Experience with the switch suggests that trainings that can disseminate guidance and build technical assistance capacity regarding OPV withdrawal should ideally begin at the global level at least a year prior to OPV withdrawal (Table 2). Trainings related to selected specific tasks, such as the OPV inventories needed to inform procurement planning, may need to begin even earlier. The global training would be followed by similar regional trainings or planning workshops and then by national and local-level trainings for immunization program staff and health workers who use OPV. Preparation of training materials will need to start at least several months beforehand. As with the switch, global materials developed by GPEI partner organizations, particularly the WHO’s Expanded Program on Immunization (EPI), can help with the development of regional, country, and local materials, although these materials will need to be adapted to local contexts. At a minimum, trainings and planning sessions should explain the rationale for OPV withdrawal, describe the steps needed to successfully execute it, and identify who is responsible for those steps and the corresponding timelines. Plans for OPV withdrawal developed at all levels will similarly need to identify the steps needed to successfully withdraw OPV, how and when to carry out those steps, and who is responsible for each step.

**Table 2.** Possible Timeline of Preparations and Activities Related to Oral Poliovirus Vaccine (OPV) Withdrawal

**Timing** ^**a**^	**Area of Work for OPV Withdrawal**					
	**General**	**Coordination**	**Communications**	**OPV Collection and Disposal**	**Monitoring**	**Financial Support**
**≥24 mo prior**	Date of global OPV withdrawal set	OPV Withdrawal Working Group assembled by GPEI World Health Assembly and other relevant bodies asked to endorse global OPV withdrawal date Manufacturers set OPV production targets through OPV withdrawal date Countries plan bivalent OPV orders in coordination with relevant procurement agency (UNICEF, Pan American Health Organization, and manufacturers) to avoid overstock	Overall communications plan developed for OPV withdrawal Ongoing communications related to IPV supply and status of polio eradication	OPV disposal practices reviewed and evaluated by GPEI in light of trivalent OPV to bivalent OPV switch experience Discussions held with vaccine manufacturers regarding OPV disposal following OPV withdrawal	OPV withdrawal incorporated into work of global and regional certification commissions All countries that will need to confirm withdrawal of OPV identified	Model developed for estimating country level OPV withdrawal costs and need for external support Country eligibility criteria for external support determined GPEI budget finalized for country-level OPV withdrawal funding Mechanism established for disbursing funds to countries
**18–24 mo prior**	Detailed global plan and guidelines developed for OPV withdrawal	Procurement planning guidelines developed for countries WHO and UNICEF regional coordinators hired for OPV withdrawal	Core reference materials for global advocacy and awareness developed on OPV withdrawal Once decision on timing is confirmed, letter on OPV withdrawal disseminated to ministers of health, including need to budget for OPV	Detailed guidelines for OPV disposal developed	Detailed global plan for monitoring OPV withdrawal developed	Broad estimate of funds for OPV withdrawal included in countries’ annual budgets Materials developed for countries to apply for external support
**12–18 mo prior**	OPV withdrawal guidance, tools, applications, and other information, including training materials on OPV disposal and withdrawal monitoring, provided from global level to regions, including through global workshop on planning for OPV withdrawal	National OPV withdrawal coordinators identified and OPV withdrawal coordination committees established Continued coordination with manufacturers and countries regarding OPV production and distribution	Full package of communications guidance and materials developed and disseminated	Countries conduct OPV inventories	…	Mechanism established for reviewing requests for external support
**7–12 mo prior**	Regional workshops for national OPV withdrawal coordination staff on planning for OPV withdrawal, including OPV disposal and monitoring OPV withdrawal OPV withdrawal guidance, tools, applications, and other information provided from regional level to countries	National OPV withdrawal plans developed Continued coordination with manufacturers and country vaccine procurement officials	National media planning Outreach begins to health professional organizations and key stakeholders, CSOs, NGOs, and others	National OPV disposal plans developed as part of OPV withdrawal plans Agreements with private sector disposal contractors established if necessary	National OPV withdrawal monitoring plans developed in conjunction with overall OPV withdrawal and disposal plans	Detailed national budgets for OPV withdrawal and disposal developed Countries’ requests for external support submitted to and reviewed by GPEI External support funds disbursed to countries
**6 wk–6 mo prior**	National materials and documents for OPV withdrawal, including OPV disposal and withdrawal monitoring, developed and printed	Subnational OPV withdrawal coordinators identified Trainings held for national and subnational immunization staff All sites involved with OPV withdrawal identified Last distribution of OPV to countries	Global, regional, and national media outreach begins Outreach begins to private sector immunization providers	Trainings held for staff involved with OPV disposal Sites for OPV disposal identified Countries conduct OPV inventories OPV stocks redistributed and OPV use maximized to minimize the amount of OPV on hand by OPV withdrawal date	NOWCC established National and subnational OPV withdrawal monitoring coordinators identified Sites to be monitored for OPV withdrawal identified	…
**2–6 wk prior**	…	Trainings held for remaining national and subnational immunization staff	Global, regional, and national media outreach continues	Trainings held for remaining staff involved with OPV disposal	Monitors for OPV withdrawal recruited	
**0–14 d prior**	…	OPV withdrawn from cold chain stores and health facilities	Global, regional, and national media outreach continues Media monitoring begins	…	Monitors for OPV withdrawal trained and equipped	Private sector health facilities compensated for unexpired OPV if necessary
**0 d–4 wk after**	…	Problems with OPV withdrawal identified by monitors addressed	Issues management plans implemented in response to any problems with OPV withdrawal	OPV disposed of Problems with OPV disposal identified by monitors addressed	Visits to cold chain stores, selected health facilities, and disposal sites by monitors NOWCCs review reports from monitors and other sources NOWCCs submit initial OPV withdrawal report to government and the WHO	…
**1–3 mo after**	…	Problems with OPV withdrawal identified by monitors addressed	Formal evaluation of OPV withdrawal communications efforts	Problems with OPV disposal identified by monitors addressed	Visits to remaining health facilities and any disposal or cold chain sites needing repeat visits NOWCCs review reports from immunization staff and other sources NOWCCs submit final OPV withdrawal reports to government and the WHO	…
**≥3 mo after**	…	Problems with continued use or storage of OPV addressed	…	…	Global Polio Laboratory Network monitors for use of OPV Immunization program staff and supervisors alert for any remaining OPV	Financial reports on external support funds submitted to GPEI by countries Recall of unspent external support funds

**Abbreviations: CSO, civil society organization; GPEI, Global Polio Eradication Initiative; NGO, nongovernmental organization**; NOWCC, National OPV Withdrawal Certification Committee; UNICEF, United Nations Children’s Fund; WHO, World Health Organization.

^**a**^ **Values indicate timing of completion of work relative to the global OPV withdrawal date.**

Several measures not undertaken for the switch could potentially aid in ensuring that use of all bOPV stops at the time of OPV withdrawal. Expiration dates of bOPV manufactured after the date of OPV withdrawal has been set could be tied to the withdrawal date, regulators could revoke the licensure of bOPV but not mOPV, or the packaging of bOPV could be altered to facilitate its tracking, perhaps through the integration of electronic devices into the label, barcode, or vial. However, all of these measures would require a great deal of cooperation from vaccine manufacturers and regulators that may not be forthcoming, and efforts to implement any of them would need to start long before the date of OPV withdrawal. Great care would also be needed to ensure that these measures did not result in any unintended problems.

## WITHDRAWAL OF OPV

Even if the amount of bOPV remaining on the date of OPV withdrawal is successfully minimized, it will still be important to remove any remaining OPV vials from the cold chain to preclude its use after the withdrawal date. While use of bOPV within a few weeks or even months of the date of general OPV withdrawal is unlikely to lead to the emergence of new cVDPVs, especially if population immunity to type 1 and 3 poliovirus infection is high at the time of OPV withdrawal [10, 13], its continued storage in the cold chain and intentional or accidental use long after OPV withdrawal would be more problematic [11]. Fortunately, the logistics of removing all OPV vials from the cold chain will likely be simpler than those of the switch. For example, the possibility of health workers confusing tOPV and bOPV was a serious concern during the switch but will not be an issue for full OPV withdrawal. These simpler logistics suggest that tight synchronization of OPV withdrawal is possible.

In the case of the switch, aspirational goals were set, to complete the switch globally within 2 weeks and to have individual countries select a single day on which all health facilities stopped use of tOPV and started use of bOPV and health staff withdrew tOPV from storage nationwide. These goals were logistically challenging, but aiming for them proved effective in terms of global synchronization [8]. Of the 150 countries and territories using OPV as of April 2016 (5 of the 155 countries and territories using OPV in 2015 ceased all routine programmatic use of OPV before April 2016), all reported ceasing tOPV use by 12 May 2016, only 11 days after the end of the official 17 April—1 May 2016 switch window [3]. A similar, aspirational goal would likely work well for OPV withdrawal, albeit with minor changes to simplify the logistics involved. For example, all health facilities and countries could be asked to stop bOPV use and withdraw all OPV not being used for outbreak responses no later than a specified global withdrawal date. Countries could be given the option of removing all OPV vials from all levels of their cold chains, starting up to 2 weeks before the global withdrawal date if other dates during that period would be more practical for them. Countries could also be given the option of actually withdrawing all OPV vials from cold chain stores over 3 days instead of just 1 day, to reduce the number of personnel and vehicles needed at one time to pick up remaining OPV vials.

## DISPOSAL OF WITHDRAWN OPV

The most certain way to ensure that OPV is not used after its withdrawal is to inactivate and destroy all OPV remaining at that time. Clear guidance that all OPV vials outside of outbreak-response stockpiles need to be destroyed after the date for OPV withdrawal will reinforce this message for immunization program staff. Confidence that all OPV vials have been destroyed outside of global stockpiles would be enhanced by careful monitoring and documentation of OPV disposal efforts, including comparisons between the amounts of OPV known to be disposed of and the amounts of OPV documented in inventories at the time of OPV withdrawal.

Guidance provided to countries by GPEI and EPI regarding OPV disposal should explain a range of options in detail that can be adapted to the policies and capabilities of individual countries. A detailed survey of countries regarding their experience with selecting and executing methods of tOPV disposal, as well as a careful literature review, could help improve the quality of the guidance provided on disposal of OPV. Ideally, any remaining OPV vials will be collected from individual cold chain stores and then inactivated and destroyed at centralized locations that can cover entire districts, provinces, or countries [21]. Such an approach would facilitate monitoring of disposal and use of relatively efficient disposal methods. While all remaining OPV vials can probably be destroyed within a month of the date of full OPV withdrawal, further research could help to better define a realistic time frame. Strong planning and preparation for transporting, inactivating, and disposing of withdrawn OPV that begins well in advance of OPV withdrawal could help countries minimize the time needed to complete the disposal of OPV.

## MONITORING AND CONFIRMING THE WITHDRAWAL OF OPV

Monitoring the withdrawal and destruction of OPV at local, country, regional, and global levels will help boost motivation for OPV withdrawal; provide an opportunity to identify, remove, and destroy any remaining OPV; and confirm that OPV will no longer be used [9]. The existence of a transparent monitoring and confirmation system will encourage the synchronization of OPV withdrawal because it will give countries more confidence that other countries will also stop using OPV [22]. Confirming full OPV withdrawal may be even more important than was confirming the completion of the switch because the stakes involved with polio outbreaks that occur after OPV withdrawal will be higher.

In many ways, the monitoring and validation of the switch provide a model for monitoring and confirming the completeness of efforts to withdraw and destroy OPV [3, 9]. For tOPV withdrawal, monitors visited all vaccine stores from the national to the district levels, as well as a purposively selected sample of high-risk health facilities, but monitors did not assess tOPV disposal sites. A validation committee reviewed the monitors’ findings in each country and assessed whether tOPV had been fully removed from the supply chain, but it did not assess whether the tOPV had been disposed of after removal. The national government received the validation committee’s assessment and transmitted it to the WHO, ideally within 2 weeks of that country’s official cessation of tOPV use. Of the 155 countries asked to provide switch validation reports to WHO, 147 (95%) provided them within a month of the last day of the switch window [9]. Countries in the Americas took an even more stringent approach by having national immunization program supervisors visit all health facilities and by having the Regional Certification Commission of the Americas review countries’ validation reports and ask for additional information or corrective measures if needed [23].

Although the vast majority of tOPV was withdrawn as planned during the switch [9], developments since the switch have indicated possible areas for improvements regarding monitoring and confirmation of OPV withdrawal. National immunization program supervisors in multiple countries in the Americas found tOPV at multiple health facilities that were not included in the sample of facilities visited by monitors, suggesting that tOPV also might have remained at facilities not visited by monitors in other regions. Expanding the proportion of health facilities visited by monitors could help reduce the likelihood of such facilities retaining OPV after its withdrawal, as could having monitors visit a representative sample of health facilities in addition to high-risk facilities, having national immunization program supervisors visit all public facilities and report on their findings, and checking reports of OPV withdrawn and destroyed against records of OPV inventories. Identifying all of the private sector end users of bOPV with the help of vaccine manufacturers, distributors, and professional organizations and then monitoring a portion of these sites could help ensure that all OPV is removed from the private sector.

In a small number of countries, the withdrawal and disposal of tOPV took significantly longer than expected, sometimes because of last-minute governmental delays [24]. Involving the Global Commission for Certification of Poliovirus Eradication and all of the related regional certification commissions in reviewing countries’ reports on their OPV withdrawal and disposal monitoring results might further encourage governments to fully withdraw and dispose of OPV in a timely manner and would position the certification commissions to assist with responses to problems with OPV withdrawal.

Although more time may be needed during OPV withdrawal than during the switch for the collection and reporting of information from visits to cold stores, health facilities, and disposal sites because of the potentially greater number of sites that will need to be visited, it will still be important to limit the time frame of the monitoring and validation phase. For example, the monitoring and validation phase could be extended from 2 weeks to 3 or 4 weeks from the date of OPV withdrawal, to better align with the amount of time most countries actually required for switch monitoring and validation. Even after the end of formal monitoring visits to confirm the withdrawal of all OPV, national immunization program supervisors should look for OPV during routine supervisory visits and remove and dispose of any they find. The Global Polio Laboratory Network will supplement the work of monitors and supervisors by performing surveillance for OPV use through its ability to detect the attenuated Sabin viruses found in OPV in environmental samples or stool samples [20]. Detection of attenuated Sabin viruses >4 months after cessation of OPV use in a given area should trigger follow-up investigations to search for any OPV still in use.

Unlike the other components of OPV withdrawal, the monitoring and confirmation process could potentially involve more than just the countries that are still using bOPV at the date of OPV withdrawal or the countries that continued to use OPV after the switch (Figure 2). To fully minimize the possibility of OPV being used after its withdrawal, all countries that have ever used OPV, including those that ceased using it years before the switch, should ideally confirm that all of their OPV has either been (1) withdrawn and destroyed or (2) safely contained in an approved poliovirus-essential facility. However, most if not all of the countries that had stopped OPV use before the time of the switch are countries with adequate sanitation and immunization schedules with multiple doses of IPV, and many of them are upper-income countries with temperate climates [25]. All of these factors reduce the likelihood that use of small amounts of OPV in these countries after OPV cessation could cause outbreaks of polio. As a result, if these countries participate in efforts to confirm that OPV has been withdrawn, much more limited searches than those undertaken in countries using OPV up until the date of its withdrawal may be appropriate.

## FINANCIAL SUPPORT FOR OPV WITHDRAWAL

Given the importance of executing and confirming OPV withdrawal in a synchronized manner, additional resources beyond what is available for routine national immunization program operations may be needed [26]. For example, additional supplies and staff may be needed for training, communications, transporting and disposing of withdrawn OPV, and monitoring and confirming the withdrawal and disposal. Despite the differences in scope, it is quite possible that similar resources will be needed overall for complete OPV withdrawal as were needed for the switch. While some areas of work, such as logistics, may cost less since there will be no distribution of new vaccine involved, others, such as monitoring, may require a more stringent effort and therefore more personnel and transport resources. As with the switch, some countries may require external financial assistance to ensure that adequate training and communications activities are completed, in addition to timely completion of OPV withdrawal. Countries that were able to use GPEI-funded staff and equipment to support the switch may need to find alternatives for OPV withdrawal since associated GPEI funding will likely have declined by that time [27]. The amount of external assistance required may be minimized if the date of OPV withdrawal is set well in advance (ie, 18–24 months) to allow national governments, local donors, and in-country partners to include the resources needed for the switch in their regular budgets. Based on the experience with the switch, some select activities, such as physical vaccine inventories to inform procurement decisions, would greatly benefit from having funding available at least 18 months prior to the date of OPV withdrawal.

If the date of OPV withdrawal and associated guidelines are not established early enough to allow countries, manufacturers, and other stakeholders to adequately plan, prepare, and budget, the amount of external resources that will be needed for successful OPV withdrawal will increase. For example, without certainty about the date of OPV withdrawal or the quantities of bOPV needed until that date, manufacturers may not produce sufficient bOPV. If there is uncertainty about bOPV supply needs when manufacturers must make decisions about how much bOPV to produce during the final period before OPV withdrawal, GPEI may need to work with manufacturers to consider options for ensuring an ample supply of bOPV. Such options many include sharing of financial risk or bulk stockpiling [13, 16]. Similarly, at a country level, GPEI may find it expedient to buy back bOPV from governments or private providers if they overstock because of uncertainty about plans for OPV withdrawal, particularly if a universal OPV expiration date is not set to coincide with the date of OPV withdrawal.

## CONCLUSIONS

In many ways, the experience with the global switch from tOPV to bOPV can help inform the eventual full global withdrawal of OPV, although the differences in context between the two and the detection of limited tOPV use after the switch suggest that some changes in procedures might be needed [8, 9, 20, 26, 28]. Given the potentially disastrous consequences of polio outbreaks caused by cVDPVs following OPV withdrawal [25, 29], it will be important to ensure that no OPV remains outside of polio-outbreak-response stockpiles or areas conducting outbreak-response campaigns. Setting a reasonably firm date for OPV withdrawal as far ahead of the withdrawal date as possible (ideally at least 24 months), involving GPEI partner support as appropriate, and beginning preparations as soon as the date is set would greatly enhance the OPV withdrawal process and would help minimize the associated costs and need for donor financial support. A shorter lead time would provide valuable flexibility for decisions about when to stop use of bOPV in the context of uncertainty about whether or not WPV1 and WPV3 had been eradicated, but it may also increase the cost of OPV withdrawal. If OPV withdrawal is thorough, comprehensive, and well synchronized, it will effectively complement other efforts underway or planned to reduce the risk from polio outbreaks from VDPVs after all wild polioviruses are eradicated. These efforts include the development of a new, extremely attenuated polio vaccine [30]; the global introduction of IPV [3, 5, 7]; the conduct of bOPV SIAs shortly before OPV withdrawal [12, 13]; the maintenance of monovalent OPV stockpiles for rapid response to polio outbreaks [3, 5]; the operation of a global polio surveillance system that can quickly detect polio outbreaks [31]; and the containment of polioviruses held in research and vaccine manufacturing facilities [32]. Taken with these other measures, the successful full withdrawal of OPV will be a key step toward ensuring a polio-free world.

## Notes

***Acknowledgments.*** We thank the participants in the September 2016 Global Polio Eradication Initiative Immunization Systems Management Group meeting in Atlanta, Georgia, for insightful discussions that helped to inform this article, and Becky Maholland, Division of Emergency Operations Situational Awareness Geographic Information Systems, Centers for Disease Control and Prevention, for the creation of the maps for the article.

***Financial support.*** This work was supported by the World Health Organization; the UNICEF; the Centers for Disease Control and Prevention; and the Task Force for Global Health, on behalf of the Bill and Melinda Gates Foundation. M. P. and J. G. were supported by the Bill and Melinda Gates Foundation (grant OPP1095024).

***Supplement sponsorship.*** This work is part of a supplement coordinated by the Task Force for Global Health with funding provided by The Bill and Melinda Gates Foundation and the Centers for Disease Control and Prevention.

***Potential conflicts of interest.*** All authors: No reported conflicts. All authors have submitted the ICMJE Form for Disclosure of Potential Conflicts of Interest. Conflicts that the editors consider relevant to the content of the manuscript have been disclosed.
